# The establishment of a WHO Reference Reagent for anti-malaria (*Plasmodium falciparum*) human serum

**DOI:** 10.1186/s12936-017-1958-x

**Published:** 2017-08-05

**Authors:** Donna Bryan, Nilupa Silva, Peter Rigsby, Thomas Dougall, Patrick Corran, Paul W. Bowyer, Mei Mei Ho, the Collaborative study group, Evelina Angov, Ed Remarque, Daniel Dodoo, Chris Drakeley, James Beeson, A. Manjurano, Simon Kariuki, Francis Ndungu, Faith Osier, Denise L. Doolan, Carlota Dobano, Benjamin Mordmueller, Ogobara Doumbo, Bourèma Kouriba, Paushali Mukherjee, Carole Long, Nilupa Silva, Donna Bryan, Mei Mei Ho, David Cavanagh

**Affiliations:** 1 Bacteriology Division, MHRA-NIBSC, South Mimms, Potters Bar, Hertfordshire, EN6 3QG UK; 2 Biostatistics Group, MHRA-NIBSC, South Mimms, Potters Bar, Hertfordshire, EN6 3QG UK

**Keywords:** *Plasmodium falciparum*, Malaria, Reference, Standardization, Serology, Vaccine, WHO Reference Reagent

## Abstract

**Background:**

At a World Health Organization (WHO) sponsored meeting it was concluded that there is an urgent need for a reference preparation that contains antibodies against malaria antigens in order to support serology studies and vaccine development. It was proposed that this reference would take the form of a lyophilized serum or plasma pool from a malaria-endemic area. In response, an immunoassay standard, comprising defibrinated human plasma has been prepared and evaluated in a collaborative study.

**Results:**

A pool of human plasma from a malaria endemic region was collected from 140 single plasma donations selected for reactivity to *Plasmodium falciparum* apical membrane antigen-1 (AMA-1) and merozoite surface proteins (MSP-1_19_, MSP-1_42_, MSP-2 and MSP-3). This pool was defibrinated, filled and freeze dried into a single batch of ampoules to yield a stable source of naturally occurring antibodies to *P. falciparum*. The preparation was evaluated by an enzyme-linked immunosorbent assay (ELISA) in a collaborative study with sixteen participants from twelve different countries. This anti-malaria human serum preparation (NIBSC Code: 10/198) was adopted by the WHO Expert Committee on Biological Standardization (ECBS) in October 2014, as the first WHO reference reagent for anti-malaria (*Plasmodium falciparum*) human serum with an assigned arbitrary unitage of 100 units (U) per ampoule.

**Conclusion:**

Analysis of the reference reagent in a collaborative study has demonstrated the benefit of this preparation for the reduction in inter- and intra-laboratory variability in ELISA. Whilst locally sourced pools are regularly use for harmonization both within and between a few laboratories, the presence of a WHO-endorsed reference reagent should enable optimal harmonization of malaria serological assays either by direct use of the reference reagent or calibration of local standards against this WHO reference. The intended uses of this reference reagent, a multivalent preparation, are (1) to allow cross-comparisons of results of vaccine trials performed in different centres/with different products; (2) to facilitate standardization and harmonization of immunological assays used in epidemiology research; and (3) to allow optimization and validation of immunological assays used in malaria vaccine development.

**Electronic supplementary material:**

The online version of this article (doi:10.1186/s12936-017-1958-x) contains supplementary material, which is available to authorized users.

## Background

Malaria is a major public health problem with 91 countries, inhabited by a total of 3.4 billion people, with on-going malaria transmission. In 2015, there were approximately 212 million cases of malaria resulting in about 429,000 deaths, most of which were children under 5 years old [1].

A working group on laboratory methods for malaria vaccines under the auspices of the World Health Organization (WHO), Initiative for Vaccine Research (IVR), PATH Malaria Vaccine Initiative (MVI) and The National Institute of Allergy and Infectious Diseases (NIAID) agreed in a meeting held in September 2005, that an immunoassay standard for *Plasmodium falciparum* malaria antibodies was urgently needed to support vaccine development particularly in immunological assays development and standardization; also for harmonization of immunological assays used in epidemiology research of malaria [2]. It was proposed that this reference material would take the form of a lyophilized serum or plasma pool from a malaria-endemic area. The anti-malaria (*P. falciparum*) human serum reference reagent was considered as important in the progress of malaria vaccine development, especially in immunological assays development and standardization. WHO ECBS endorsed this proposal in 2005.

The reference reagent contains anti-malarial antibodies for multiple antigens and malaria species, but the initial standardization exercise concentrated on antibodies to *P. falciparum* apical membrane antigen-1 (AMA-1) and merozoite surface proteins (MSPs). This manuscript describes the collection, production and evaluation of this plasma pool leading to the establishment of the first International Reference Reagent for anti-malaria (*Plasmodium falciparum*) human serum as well as a further study describing the presence of anti-CSP antibodies and the range of anti-MSP-1_19_ antibodies from different malaria species.

## Methods

### Protein antigens

*Escherichia coli*-derived: EcMSP3 (FVO), EcMSP-1_42_ (3D7), EcMSP-1_42_ (FUP), EcMSP-1_42_ (FVO) and *Pichia pastoris*-derived: Pp AMA1 (3D7), PpAMA1 (FVO), PpAMA1 (L32) PpCSP-M3 (3D7) proteins were obtained from the Malaria Vaccine Development Branch, NIAID/NIH, Rockville, MD 20852, USA.

Recombinant MSP-1_19_ from *P. falciparum*, *Plasmodium vivax*, *Plasmodium malariae* and *Plasmodium ovale* was obtained from The European Malaria Reagent Repository (http://www.malariaresearch.eu).

### Donation of plasma samples

A proposal to collect malaria reactive plasma samples was developed and approved by the ethical boards of CDC and KEMRI and the Kenyan National Blood Bank Transfusion Services (KNBTS), and agreed by the Chief Medical Officier (CMO), Kenya Ministry of Health. As a result, in 2009, 140 plasma samples (20–50 ml volume and malaria reactive) were collected, screened for HIV, HBV and HCV by the blood bank, CDC, Kisumu, and sent to NIBSC, U.K. by the KEMRI/CDC.

### Lyophilization of anti-malaria defibrinated plasma pool (10/198)

At NIBSC, the plasma samples were initially screened by ELISA against five *P. falciparum* antigens; AMA-1, MSP-1_19_, MSP-1_42_, MSP-2 and MSP-3. Samples with high titre anti-AMA-1 were pooled into four groups and defibrinated. The resulting serum was pooled, diluted 1:5 with sterile distilled water without any other excipients and filtered (1.2, 0.45 then 0.22 µm). No other buffer, bulking agent or stabilizer was used. The definitive fill was performed within the Standards Processing Division of NIBSC using a Bausch & Strobel Filling Machine (AFV5090). The diluted serum preparation was stirred constantly during filling to ensure homogeneity of fill and the temperature was maintained between 4 and 8 °C. DIN clear glass ampoules with capacity of 5 ml volume were used. Freeze-drying was performed using a 4-day cycle and the finished product was coded 10/198 and stored in the dark at −20 °C except for a small number of ampoules used for an accelerated degradation study.

A single batch of about 5000 ampoules was produced. The ampoules have an average actual fill weight of 1.0085 g per ampoule; with a target CV of precision of filling for fill mass of <0.25%, where the actual CV of fill mass was 0.11% (of 195 randomly selected ampoule, weights being measured). Nitrogen was used to back fill the freeze drier chamber at the end of the cycle and hence provide the headspace gas for the ampoules. The purity of the boiled off nitrogen was certified at 99.99%. Microbiological assessment was made on the product pre- and post-processing and the bacterial, mould, and yeast colony counts were negligible at pre- and post-filling stages. The residual moisture content of this preparation is 0.84% as determined by the Abderhalden method, with a CV of 12%. The mean oxygen head space was determined to be 0.34% with a CV of 20%. This was determined by taking a mean of 12 determinations using frequency modulation spectroscopy (FMS), a non-destructive infra-red laser absorption technique.

### Reference controls

Three additional human serum preparations (as internal controls, C1, C2 or C3) representing different titres for anti-AMA-1 and anti-MSP-2 were also filled and lyophilized to be included as test samples in the collaborative study.

### Collaborative study design

#### Participants

The candidate WHO Reference Reagent (Code: 10/198) was distributed to sixteen participating laboratories in twelve different countries (The Collaborative Study Group, see Acknowledgements). All invited participants were sent a questionnaire asking them to confirm having extensive experience in ELISA. The collaborative study involved a wide range of appropriately qualified laboratories representing clinical and research laboratories, academia, not-for-profit research organizations, government-funded organizations and charitable organizations worldwide covering Africa, Asia, Europe and the USA. For this study, a code number was allocated at random to each participant.

#### ELISA protocol for collaborative study

Each participant was provided with 4 ampoules of positive control (XC) and 4 sets of nine blinded test samples (labelled with just the sample number) comprising coded duplicates of the 10/198 (coded S1 and S2 in this study) and internal controls. C1, C2 or C3 (see Table 1). The internal controls are also malaria reactive plasma pools from Kenya that were selected to provide a range of responses by ELISA thus ensuring a breadth of relative potencies for analysis in the collaborative study. Participants were requested to test nine blinded samples and a positive control as labelled using a specific study protocol. All ELISA plates included duplicates of 10/198 which were coded as ‘S1’ (sample numbers 1, 4 and 8) or ‘S2’ (sample numbers 2, 6 and 9) for statistical analysis.

**Table 1:** Samples provided to the collaborative study participants

Sample number	Product code	Product name
Control	Pool 4	Positive control (XC)
1	10/198	Reference Reagent (S1)
2	10/198	Reference Reagent (S2)
3	Pool 2	Control 2 (C2)
4	10/198	Reference Reagent (S1)
5	12/192	Control 3 (C3)
6	10/198	Reference Reagent (S2)
7	Pool 1	Control 1 (C1)
8	10/198	Reference Reagent (S1)
9	10/198	Reference Reagent (S2)

In brief, 96-well ELISA plates (Immulon 4, flatbottom) were coated with 50 µl of antigen (2 µg/ml) in coating buffer (1.59 g Na_2_CO_3_ + 2.93 g NaHCO_3_ per litre in deionized water, pH 9.4–9.6). The coated plates were sealed and stored overnight at 4 °C. After 3 washes with PBS-0.05% Tween 20 (PBS-T) plates were blocked with 200 µl of 1% skimmed milk (w/v) in PBS-T (diluent buffer) for 3 h at room temperature. Plates were washed a further 3 times in PBS-T, followed by adding 100 µl of diluent buffer into all wells.

Test samples and controls were reconstituted in 1 ml deionized water and mixed. To Column 1, 50 µl of reconstituted test samples or XC was added in duplicate to each well. A threefold serial dilution was prepared in diluent buffer across the entire plate. Plates were sealed and incubated overnight at 4 °C.

Plates were washed 6 times in PBS-T and 100 µl HRP-conjugated rabbit anti-human IgG (DAKO, 1:5000 in diluent buffer) was added before incubation for 3 h at room temperature. Plates were washed a further 6 times before colour development by addition of 100 µl/well of OPD substrate solution prepared as per the manufacturers instruction. The reaction was stopped by addition of 25 µl/well 2 M H_2_SO_4_ and absorbance at 492 nm was measured using a plate reader.

Participants were asked to perform three independent experiments on each set of samples, for three different coating antigens, using freshly reconstituted ampoules on each experimental day. For each experimental day a total of 27 antigen-coated plates were required for the three different antigens (MSP3, MSP-1_42_ and AMA-1 3D7; i.e. 9 plates per antigen). The plate layout was such that each ELISA plate would generate results for two individual samples of 10/198 (S1 and S2), the positive control (XC) and an internal control (C1, C2 or C3).

All raw data from participants were sent and collated at NIBSC for statistical analysis.

### Stability studies

The stability studies were performed at NIBSC only. Two independent ELISA experiments (as above) were performed for each antigen; three individual ampoules of each sample group were tested for each experiment.

Accelerated thermal degradation: ampoules of the 10/198 were stored at −150, −70, −20, 4, 20, 37, 45, or 56 °C, for 1, 3, 6, 9, 12, 18, or 24 months. At each time point the ampoules stored at the various temperatures were transferred to storage at −20 °C until further analysis. The stability samples (stored at various temperatures and at the fixed time points) were reconstituted and assessed using the same ELISA method described as above and compared to 10/198 stored at −150 °C.

In use stability after reconstitution: Ampoules were reconstituted in deionized water and stored at 4 or −20 °C for 1 or 3 months. After storage the samples were analysed and compared to freshly opened and reconstituted samples in the ELISA assay.

### Statistical analysis

The potencies of all samples were calculated relative to 10/198 (S1) for each antigen from each participant laboratory by analysis of the raw optical density assay data. Log transformed absorbance values were used as the assay response in all cases. As a large volume of data of varying quality was received, full curves were not fitted and analysis was based only on an approximately linear section of the response range by excluding serum dilutions with responses falling outside 20–80% of the response range observed on the plate. Where this gave fewer than three dilutions for analysis the sample was excluded. As this resulted in an excessive number of exclusions in laboratories 3, 10 and 15, the response range was extended to 10–90% for these laboratories.

The assays were analysed with a weighted logistic parallel line model, using custom software, WRANL [3]. Assays where visual assessment of the plotted data clearly indicated that a parallel line model would not fit the data were excluded from further analysis. Assay validity was assessed by calculation of the ratio of slopes for each test sample relative to S1. The samples were concluded to be non-parallel when the slope ratio was outside of the range 0.67–1.50 and no estimates were reported. Where potency estimates were calculated, EC_50_ estimates (concentration required to achieve a 50% response) were also determined in order to assess their variability within and between laboratories.

Relative potency and EC_50_ estimates from all valid assays were combined to generate an unweighted geometric mean (GM) for each laboratory and these laboratory means were used to calculate an overall unweighted geometric mean for each sample. Variability between assays within laboratories and between laboratories has been expressed using geometric coefficients of variation (${\text{GCV}} = \{ 10^{s} - 1\} \times 100\%$ where *s* is the standard deviation of the log_10_ transformed estimates). Analysis of variance with Duncan’s multiple range test [4] using the log transformed potency estimates was used to compare laboratories and samples (p < 0.05 used to conclude significance).

### Cross reactivity with other antigens

The cross reactivity of 10/198 with circumsporozoite protein (CSP) or MSP-1_19_ from different malaria species (*P. falciparum*, *P. vivax*, *P. ovale* or *P. malariae*) was investigated by ELISA (as above with few modifications). These experiments were performed at NIBSC only.

*CSP ELISA*; ELISA plates were coated at 2 µg/ml PpCSP in coating buffer overnight at 4 °C. Caesin (1% w/v in PBS-T) was used as the blocking agent. TMB (100 µl) was used as peroxidase substrate for colour reaction which was stopped after 20 min by addition of 50 µl 1 M H_2_SO_4_. Plates were read on a Multiskan EX plate reader (Thermofisher) in absorbance mode at 450 and 620 nm. The absorbance background values at 620 nm were subtracted from those at 450 nm. In addition OD from negative control wells (no antigen) were subtracted from the OD of experimental samples.

In addition the intended use of 10/198 was demonstrated for potency evaluation of several test samples. These additional test samples (sample 23, 25 or 34) were from malaria reactive plasma packs stored at NIBSC. The data were analysed using CombiStats ver 5.0 (http://combistats.edqm.eu/) with a sigmoid curves model to determine potencies of the test samples relative to 10/198.

*Competitive MSP-1*_*19*_ *ELISA* was conducted similar to previous reports for mixed AMA-1 alleles [5] and was divided into two stages using 2 µg/ml of species specific recombinant MSP-1_19_ (Pf, Pm, Pv, Po) as coating antigens. In Stage one, a tritration of 10/198 was conducted to establish an optimal dilution factor that achieves a response in the approximately linear region of the dose response range (ca. 20–80%). This optimal dilution factor of 10/198 was then used in Stage two ELISA in the presence of the four recombinant MSP-1_19_s (at various concentrations). In brief, the plate was coated and blocked as before (2 µg/ml antigen) and then varying concentrations of competitor MSP-1_19_ (50 µl) was added in twofold dilution across a 96-well plate to deliver a highest concentration of 30 µg/ml MSP-1_19_ (from each of the species). This was followed by the 10/198 at the optimal dilution determined in stage one. The remainder procedure of the ELISA was performed as previously described.

## Results and discussion

### Data returned from study participants

The analysis of 10/198 was conducted by 16 different laboratories, data from a total of 1227 ELISA plates were received for statistical analysis (409 per antigen type). Data from experiment 1 for Laboratory 3 were not analysed as the substrate used was not consistent with that used in experiments 2 and 3. No other such inconsistencies were reported. Analysis of the remaining assays as described above gave valid estimates of relative potency in 93.6, 92.1 and 92.7% of cases for AMA-1, MSP-1_42_ and MSP-3, respectively.

### Relative potencies of S1 and S2

Relative potency estimates for the two individual ampoules of 10/198 (S1 and S2) are summarized in Additional file 1: Table S1. The agreement between potency estimates for S1 and S2 within plates can be assessed using the intra-laboratory geometric coefficients of variation (GCVs). These represent the variability between assays of direct comparisons of the duplicate samples. They range from 4.9% (Laboratory 16, MSP-1_42_ antigen), showing good agreement, to 73.2% (Laboratory 10, AMA-1 antigen), showing a high level of variability. In the majority of cases, GCVs were generally less than 30%.

### Analysis of potency relative to 10/198 reduces inter laboratory variation

Analysis of the log potencies by Duncan’s multiple range test was used to determine any ‘outlier’ laboratories by indicating cases where a laboratory’s estimates differed significantly from those obtained in all other laboratories. This was the case for Laboratory 2 (sample XC, all antigens), Laboratory 3 (sample C1, MSP-3 antigen), Laboratory 4 (sample XC, all antigens; sample C3, AMA-1 and MSP-1_42_ antigens) and Laboratory 13 (sample XC, MSP-3 antigen; sample C1, AMA-1 antigen; sample C2, AMA-1 and MSP-1_42_ antigens). All overall means and GCVs are therefore shown both including and excluding Laboratories 2, 3, 4 and 13 (Additional file 1: Tables S2–S7).

Table 2 summarizes the inter-laboratory variations after exclusion of outliers and demonstrates the benefits of determining relative potency rather than using EC_50_ as an absolute measure of potency. The observed GCVs are higher if EC_50_ data were considered with a range of 46.6–82.7%. However, when relative potency (to reference reagent 10/198) is calculated the GCVs reduced considerably to 5.4–13.9%. Without the exclusion of outliers the same trend remains (GCV range 46.6–82.7% for EC_50_, 9.5–55.6% for relative potency, Additional file 1: Tables S1–S7). Similarly to the inter laboratory data there is a reduction in the intra-laboratory variability when relative potency is used in place of EC_50_, albeit a smaller one.

**Table 2:** Potency (relative to S1) and EC_50_ estimates for samples XC, C1, C2 and C3

Antigen	Sample			
	XC	C1	C2	C3
*A. Potency relative to S1: Between-laboratory geometric mean and geometric coefficient of variation (%) after exclusion of laboratories 2, 3, 4 and 13*				
AMA-1	0.809 (9.6%)	3.549 (8.2%)	0.870 (5.7%)	0.014 (12.5%)
MSP-1_42_	0.493 (8.3%)	2.447 (8.4%)	0.871 (6.6%)	0.033 (11.5%)
MSP-3	0.548 (7.9%)	3.026 (6.2%)	0.812 (5.4%)	0.041 (13.9%)
*B. EC*_*50*_ *estimates: Between-laboratory geometric mean and geometric coefficient of variation (%) after exclusion of laboratories 2, 3, 4 and 13*				
AMA-1	0.071 (79.7%)	0.016 (62.4%)	0.069 (69.5%)	3.849 (82.7%)
MSP-1_42_	0.309 (52.8%)	0.061 (48.1%)	0.167 (56.0%)	4.406 (46.6%)
MSP-3	0.620 (60.6%)	0.108 (63.9%)	0.441 (53.6%)	7.892 (62.0%)

**Fig. 1:**
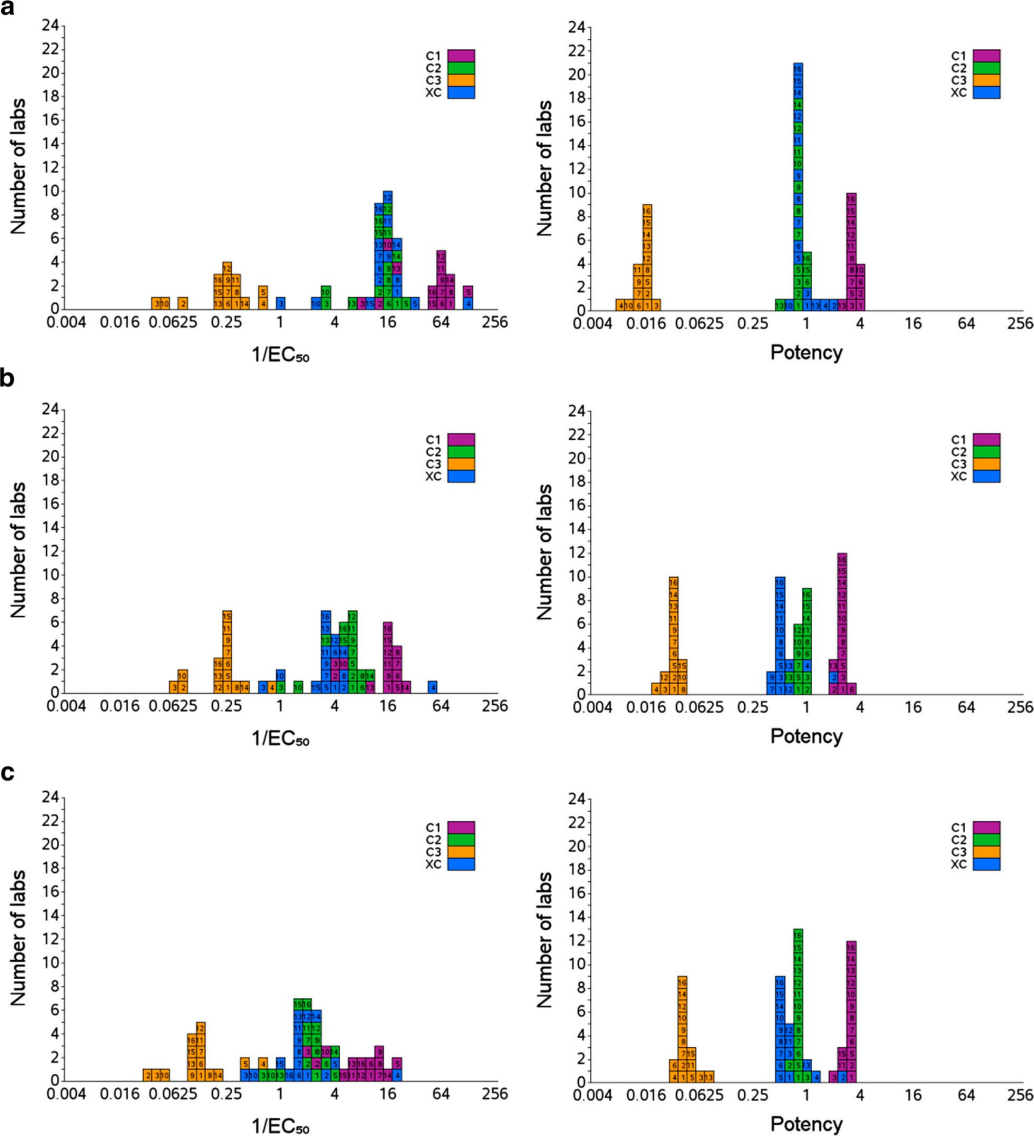
Inter-laboratory variability is reduced when potency relative to the reference reagent 10/198 replaces direct measurement. Box histograms show ELISA derived values for 1/EC_50_ (*left*) and potency (*right*) for samples C1, C2, C3 and XC from each laboratory (1–16) with AMA-1 as the coating antigen (**a**), MSP-1_42_ as the coating antigen (**b**), and MSP-3 (**c**)

Figure 1 shows box-histograms of the observed relative potency (when 10/198 is used as a reference reagent) and 1/EC_50_ for the four test samples (C1, C2, C3 and XC). In each case a reduction in the inter laboratory variations can be seen when relative potency is reported instead of 1/EC_50_. These data demonstrate the benefit of using 10/198 as a common reference reagent across assays and laboratories.

### Reference reagent 10/198 is stable at elevated temperature and after reconstitution

**Fig. 2:**
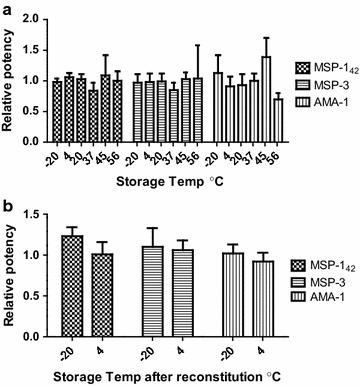
Reference reagent 10/198 is stable at elevated temperature for 24 months and after reconstitution. **a** Relative potency measured by ELISA, compared to storage at −150 °C, after 24 months storage. **b** Relative potency measured by ELISA, compared to freshly reconstituted 10/198, after storage of reconstituted content for 3 months

The reference reagent 10/198 has been lyophilized to maximize the long-term stability for storage and shipment. The thermal stability was assessed in a degradation study at elevated temperatures over a range of time points. The 24 month samples were analysed at NIBSC using the same ELISA protocol as in the collaborative study. The samples were readily reconstituted within 1 min, except for the ampoules stored at 56 °C, which required approximately 1–2 min instead to be fully reconstituted. Geometric mean potency estimates of samples stored at different temperatures for 24 months (expressed relative to those stored at −150 °C) are shown in Fig. 2a. Statistical analysis indicated that there was no detectable loss of reactivity even at 45 °C for 24 months, for AMA-1, MSP-1_42_ or MSP-3. At 56 °C for both MSP-1_42_ and MSP-3 no loss of activity was seen. Based on these data there was insufficient degradation to predict a yearly loss for 10/198.

In use stability after reconstitution in deionized water was assessed at −20 or 4 °C for 1 or 3 months. By comparison to freshly reconstituted preparation there is no evidence of any significant loss in relative potency of reconstituted content after storage for 3 months (Fig. 2b).

The use of Reference Reagent 10/198 as a standard for the calibration of serology assays has resulted in a significant decrease in inter and intra-laboratory variations and thus demonstrates the benefit of using this reagent for standardization of *P. falciparum* serological experiments. Furthermore the lyophilized preparation shows no loss in reactivity under accelerated degradation conditions and is stable for up to 3 months after reconstitution at 4 °C or lower. The preparation is maintained under controlled storage conditions at NIBSC on behalf of WHO and is ready for worldwide distribution when requested.

### Unitage proposal

There is currently no WHO International Standard/Reference Reagent of human antiserum for any species of malaria. As there is no suitable preparation with known unitage for the calibration of 10/198, it was proposed to WHO ECBS, and agreed by participants, that this the first WHO Reference Reagent for Anti-Malaria (*P. falciparum*) human serum contains an arbitrary unitage of 100 Units per ampoule.

### Reference reagent 10/198 recognizes CSP of *Plasmodium falciparum*

The collaborative study was designed to test the suitability of 10/198 to act as a reference reagent for serology studies against *P. falciparum* antigens. The study assessed the binding of the reference serum to the merozoite antigens AMA-1, MSP-1_42_ and MSP3. However, the most advanced malaria vaccine candidate RTS,S is targeted against the pre-erythrocytic antigen, CSP. In July 2015 the vaccine candidate RTS,S/AS01 received a positive scientific opinion from the European Medicines Agency for the prevention of malaria in children aged 6 weeks to 17 months in sub-Saharan Africa [5].

**Fig. 3:**
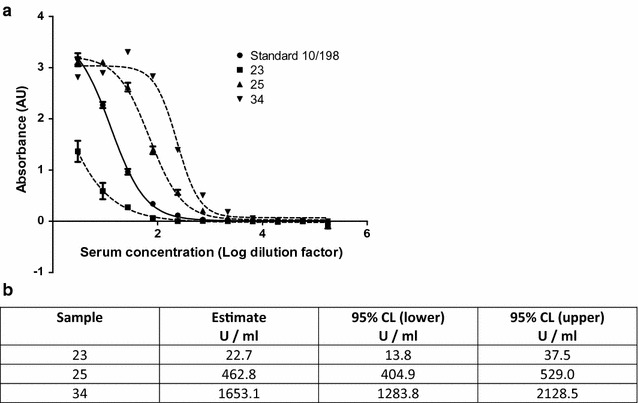
Reference reagent 10/198 detects recombinant PpCSP in ELISA. Serum dilution (Log) vs absorbance (AU) was measured for 10/198 and three unknown samples by ELISA with PpCSP as the coating antigen. Each ampoule of 10/198 contains 100 Units as assigned unitage. Parallel line analysis of the data presented in **a** was used to derive the unitage assigned in test samples (**b**)

Therefore, a separate investigation at NIBSC sought to determine if 10/198 could act as a reference reagent for detecting other malarial antigens. The reactivity of 10/198 to a recombinant CSP produced in *Pichia* was evaluated. Figure 3 shows that 10/198 is able to recognize CSP in a similar ELISA protocol as used in the collaborative study and, as a demonstration, this reference reagent was used to assign units to three plasma samples based on the designation of 10/198. Figure 3 shows sample 23 had a relative potency of 22.7 U/ml (95% CL 13.8–37.5), considerably less potent than 10/198 while samples 25 and 34 had values of 462.8 (95% CL 404.9–529.0) and 1653.1 (95% CL 1283.8–2128.5) U/ml. These data demonstrate that 10/198 also contains antibodies recognizing CSP and could be of beneficial as a reference reagent beyond the scope of the antigens tested in the collaborative study.

### Reference plasma 10/198 binds to PmMSP1_19_ and PoMSP1_19_ but not PvMSP1_19_

**Fig. 4:**
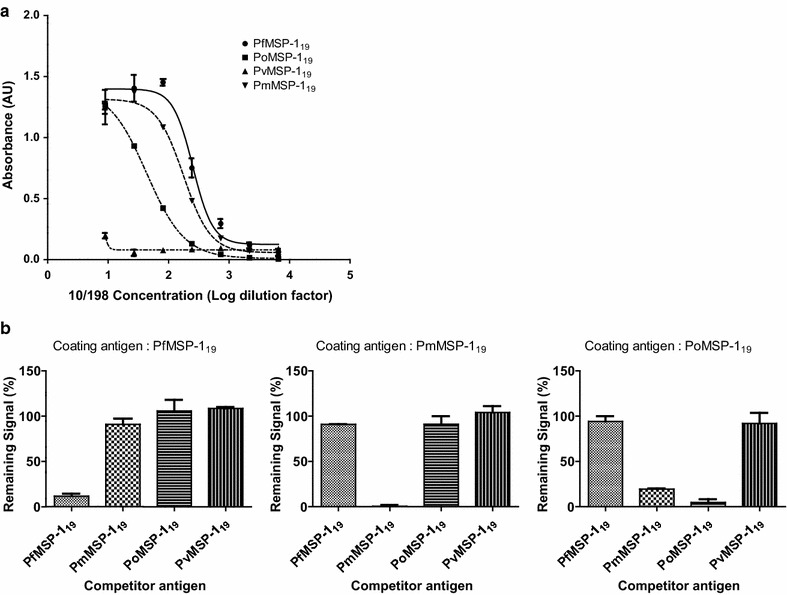
Reference reagent 10/198 also contains antibodies that recognize to *P. malariae* and *P. ovale* MSP-1_19_ but not *P. vivax*. **a** Absorbance (AU) resulting from ELISA measuring titration of 10/198 against different coating antigens at 2 µg/ml; MSP-1_19_ from *P. falciparum*, *P. malariae*, *P. ovale* or *P. vivax.* **b** Residual signal, after competition of 10/198 with 30 µg/ ml of antigen, for each of the different coating antigens

It was also expected that 10/198, which was prepared from a pool of plasma samples from a malaria-endemic area, may contain antibodies from malaria species other than *P. falciparum*. To investigate this, ELISA was performed using recombinant MSP-1_19_ from each of *P. falciparum*, *P. vivax*, *P. malariae* and *P. ovale*. The relative titre of antibodies to each of these antigens is shown in Fig. 4. These data indicate 10/198 has significant reactivity with *P. malariae* MSP-1_19_ and *P. ovale* MSP-1_19_ but no significant reactivity with *P. vivax* MSP-1_19_ as could be expected with plasma samples collected in East Africa. Competitive ELISA (Fig. 4b) was used to investigate the species specificity of the binding to each individual MSP-1_19_. Only PfMSP-1_19_ (at 30 µg/ml) was able to compete for binding when using PfMSP-1_19_ as the coating antigen; with similar specificity for PmMSP-1_19_ as evidenced by the residual signal reducing to nearly zero when only the homologous competitor was used. Some mixed competition was observed when *P. ovale* MSP-1_19_ was used as the coating antigen with both PmMSP-1_19_ and PoMSP-1_19_ able to compete at 30 µg/ml. As a control for PvMSP-1_19_ a lyophilized serum standard (71/281) was used, that was derived from a blood transfusion initially diagnosed as *P. vivax* malaria, but not characterized further (Additional file 1: Figure S1). These data demonstrate that the reference reagent 10/198 contains antibodies capable of recognizing MSP-1_19_ from *P. malariae* and *P. ovale* as well as *P. falciparum*. This is not unexpected given the reference reagent is derived from a pool of malaria reactive samples from adults but with no additional knowledge of prior malaria exposure this cannot be unequivocally linked to *P. malariae* or *P. ovale* infection.

## Conclusions

In October 2014, the WHO ECBS adopted this preparation (10/198) as the first WHO Reference Reagent of anti-malaria (*Plasmodium falciparum*) human serum with assigned an arbitrary unitage of 100 units (U) per ampoule [6, 7]. It is stored at NIBSC (Code Number 10/198) for distribution worldwide since 2015. This reference reagent should be particularly useful for inter-laboratory standardization and harmonization of immunological assays used in vaccine development and epidemiology. Analysis of reagent requests suggests a number of manufacturers and academic research groups are making use of the 10/198. It is expected that these are predominantly in the field of sero-epidemiology and diagnostics development potentially reflecting a change from the expected primary use conceived at the initiation of this project.

This reference reagent, whilst evaluated in the collaborative study against a few selected antigens, is expected to be constitute a valuable reagent over a far wider range of antigens including CSP, the antigen in the malaria vaccine, Mosquirix. However, testing has revealed no significant levels of antibodies against Rh5, a leading blood stage vaccine candidate (Silk and Draper pers. comm). The establishment of the first WHO Reference Reagent of anti-malaria (*P. falciparum*) human serum represents a pool of serum from Kenyan adults and is intended to act as a reference point for serological assays. It is expected that local, more geographically relevant, pools may be calibrated against this reference reagent as part of a broader standardization process.

In addition to use in serology assays this reagent represents a standard reflecting the antibody composition consistent with naturally acquired immunity and it is expected that it will be beneficial in the standardization of a range of functional immunoassays. Osier et al. recently demonstrated the activity of pooled human IgG from malaria exposed Kenyan adults in an opsonic phagocytosis assay [8], although there appears to be little growth inhibitory activity for 10/198, when reconstituted and applied to standard in vitro culture without further manipulation (Bowyer and Khatri unpublished data).

## Additional file

**Additional file 1.** Supplemental figure and tables.
