# Supplementary material for: The establishment of a WHO Reference Reagent for anti-malaria (Plasmodium falciparum) human serum
Source: Malar J. 2017 Aug 5;16:314. doi: 10.1186/s12936-017-1958-x (PMC5545088; doi:10.1186/s12936-017-1958-x)
Supplement: Supplementary file 1 — Additional file 1. Supplemental figure and tables. [file 12936_2017_1958_MOESM1_ESM.docx]

**Figure S1: PvMSP-1_19_ is recognised by serum 71/281**

71/281, a lyophilised serum containing antibodies to *Plasmodium vivax*, when analysed for binding to PvMSP-1_19_ can be competed by PvMSP-1_19_ but not PfMSP-1_19,_ PmMSP-1_19_ or PoMSP-1_19_.

[A] Dose response curve for serial dilution of 71/281 binding to PvMSP-1_19_

[B] Competitive ELISA using 30µg/ml competitor antigen with PvMSP-1_19_ as coating antigen.

Table S1 **Relative potencies of two individual samples (S1 and S2) of candidate 10/198**.

| **Lab** | **AMA1** | | | **MSP1** | | | **MSP3** | | |
| --- | --- | --- | --- | --- | --- | --- | --- | --- | --- |
|  | **GM** | **GCV** | **N** | **GM** | **GCV** | **N** | **GM** | **GCV** | **N** |
| 1 | 1.061 | 18.0 | 24 | 1.090 | 18.0 | 25 | 1.026 | 15.8 | 27 |
| 2 | 1.083 | 30.8 | 25 | 1.130 | 27.6 | 25 | 1.000 | 48.7 | 24 |
| 3 | 1.021 | 25.1 | 10 | 1.108 | 61.5 | 9 | 1.032 | 33.2 | 12 |
| 4 | 1.142 | 11.0 | 3 | 0.878 | 12.1 | 2 | 1.026 | 14.0 | 3 |
| 5 | 1.044 | 15.3 | 27 | 1.032 | 16.5 | 25 | 1.029 | 14.1 | 27 |
| 6 | 1.048 | 31.4 | 27 | 1.055 | 20.3 | 25 | 1.035 | 25.4 | 23 |
| 7 | 1.020 | 12.2 | 27 | 1.004 | 8.3 | 27 | 0.962 | 14.1 | 26 |
| 8 | 1.049 | 16.6 | 26 | 1.045 | 18.1 | 26 | 1.035 | 21.4 | 26 |
| 9 | 1.050 | 9.3 | 26 | 0.979 | 19.9 | 27 | 0.998 | 18.1 | 26 |
| 10 | 0.860 | 73.2 | 23 | 1.045 | 27.9 | 22 | 0.996 | 53.1 | 20 |
| 11 | 1.000 | 11.1 | 27 | 1.032 | 12.8 | 24 | 0.990 | 35.2 | 26 |
| 12 | 0.993 | 24.0 | 26 | 0.971 | 25.9 | 27 | 0.928 | 25.7 | 25 |
| 13 | 0.947 | 52.7 | 26 | 0.955 | 25.7 | 23 | 1.195 | 61.7 | 26 |
| 14 | 0.970 | 18.3 | 27 | 1.001 | 14.7 | 26 | 1.023 | 12.8 | 27 |
| 15 | 0.949 | 35.5 | 27 | 0.962 | 17.8 | 26 | 0.984 | 9.8 | 27 |
| 16 | 0.999 | 6.9 | 27 | 0.985 | 4.9 | 27 | 0.992 | 5.2 | 27 |
|  |  |  |  |  |  |  |  |  |  |

Key:

The geometric mean (GM) and geometric coefficient of variation (GCV) within each laboratory for the relative potency of S2 relative to S1 is stated. N is the number of estimates used in calculation of mean from each laboratory.

Table S2 - Relative potencies of samples XC, C1, C2 and C3 relative to candidate 10/198 (S1) in assays using AMA-1 as coating antigen

| **Lab** | **XC** | | | **C1** | | | **C2** | | | **C3** | | |
| --- | --- | --- | --- | --- | --- | --- | --- | --- | --- | --- | --- | --- |
|  | **GM** | **GCV** | **N** | **GM** | **GCV** | **N** | **GM** | **GCV** | **N** | **GM** | **GCV** | **N** |
| 1 | 0.952 | 23.1 | 24 | 4.366 | 41.4 | 8 | 0.885 | 15.3 | 7 | 0.016 | 32.8 | 9 |
| 2 | 1.981 | 54.0 | 24 | 3.777 | 23.4 | 7 | 0.852 | 18.7 | 9 | 0.017 | 41.5 | 9 |
| 3 | 1.100 | 49.8 | 9 | 3.458 | 25.2 | 3 | 0.759 | 22.6 | 2 | 0.021 | . | 1 |
| 4 | 1.768 | 92.3 | 4 | . | . | . | . | . | . | 0.008 | 49.3 | 4 |
| 5 | 0.809 | 15.5 | 26 | 3.496 | 23.2 | 9 | 0.851 | 5.2 | 8 | 0.015 | 18.6 | 9 |
| 6 | 0.720 | 48.6 | 27 | 3.673 | 31.5 | 9 | 0.953 | 12.2 | 9 | 0.013 | 24.0 | 9 |
| 7 | 0.791 | 16.3 | 27 | 3.365 | 13.4 | 9 | 0.891 | 10.7 | 9 | 0.014 | 18.7 | 9 |
| 8 | 0.840 | 16.6 | 25 | 3.361 | 11.4 | 8 | 0.863 | 8.9 | 9 | 0.014 | 21.1 | 9 |
| 9 | 0.830 | 14.0 | 27 | 3.534 | 8.9 | 9 | 0.830 | 24.9 | 9 | 0.014 | 14.9 | 9 |
| 10 | 0.657 | 55.7 | 21 | 3.868 | 66.5 | 5 | 0.834 | 43.5 | 7 | 0.011 | 3.9 | 5 |
| 11 | 0.812 | 15.4 | 27 | 3.324 | 8.0 | 9 | 0.844 | 15.8 | 9 | 0.013 | 13.9 | 9 |
| 12 | 0.874 | 17.7 | 27 | 3.367 | 26.8 | 9 | 0.871 | 10.5 | 8 | 0.015 | 36.0 | 9 |
| 13 | 1.258 | 92.9 | 26 | 2.564 | 36.8 | 9 | 0.521 | 74.8 | 9 | 0.017 | 17.6 | 8 |
| 14 | 0.803 | 17.2 | 27 | 3.539 | 14.2 | 9 | 0.783 | 11.9 | 9 | 0.015 | 13.3 | 9 |
| 15 | 0.812 | 18.3 | 27 | 3.465 | 29.6 | 9 | 0.944 | 13.8 | 9 | 0.017 | 9.6 | 9 |
| 16 | 0.839 | 5.5 | 27 | 3.361 | 4.7 | 9 | 0.899 | 3.1 | 9 | 0.015 | 8.9 | 9 |
| Overall  GM | 0.941 | | | 3.482 | | | 0.832 | | | 0.014 | | |
| Between-lab  GCV | 36.2 | | | 11.8 | | | 15.3 | | | 23.2 | | |
| Overall  GM* | 0.809 | | | 3.549 | | | 0.870 | | | 0.014 | | |
| Between-lab  GCV* | 9.6 | | | 8.2 | | | 5.7 | | | 12.5 | | |

Key:

GM – geometric mean

GCV – geometric coefficient of variation (%)

N – number of estimates used in calculation of mean

*excludes Laboratories 2, 3, 4 and 13

Table S3 Relative potencies of samples XC, C1, C2 and C3 relative to candidate 10/198 (S1) in assays using MSP-1_42_ as coating antigen

| **Lab** | **XC** | | | **C1** | | | **C2** | | | **C3** | | |
| --- | --- | --- | --- | --- | --- | --- | --- | --- | --- | --- | --- | --- |
|  | **GM** | **GCV** | **N** | **GM** | **GCV** | **N** | **GM** | **GCV** | **N** | **GM** | **GCV** | **N** |
| 1 | 0.521 | 20.7 | 25 | 2.468 | 15.7 | 8 | 0.871 | 11.4 | 9 | 0.032 | 24.3 | 8 |
| 2 | 1.951 | 56.0 | 25 | 2.218 | 45.2 | 9 | 0.979 | 51.8 | 7 | 0.033 | 10.0 | 9 |
| 3 | 0.556 | 57.3 | 8 | 2.807 | 49.6 | 3 | 1.121 | 49.0 | 4 | 0.026 | . | 1 |
| 4 | 0.995 | 94.3 | 4 | . | . | . | . | . | . | 0.019 | 6.9 | 3 |
| 5 | 0.472 | 20.7 | 26 | 2.813 | 36.8 | 8 | 0.858 | 24.2 | 9 | 0.033 | 13.8 | 8 |
| 6 | 0.500 | 14.9 | 26 | 2.904 | 21.9 | 9 | 0.989 | 21.4 | 8 | 0.031 | 25.5 | 9 |
| 7 | 0.431 | 20.9 | 27 | 2.466 | 15.8 | 9 | 0.835 | 13.9 | 9 | 0.030 | 32.7 | 9 |
| 8 | 0.512 | 22.7 | 26 | 2.241 | 16.0 | 9 | 0.991 | 25.1 | 8 | 0.036 | 17.7 | 9 |
| 9 | 0.438 | 18.6 | 27 | 2.361 | 18.5 | 9 | 0.876 | 17.6 | 8 | 0.030 | 10.8 | 9 |
| 10 | 0.473 | 32.5 | 23 | 2.429 | 20.3 | 7 | 0.846 | 48.6 | 7 | 0.041 | 44.3 | 8 |
| 11 | 0.479 | 14.3 | 24 | 2.272 | 11.7 | 7 | 0.917 | 20.3 | 8 | 0.034 | 10.2 | 9 |
| 12 | 0.567 | 34.6 | 26 | 2.507 | 17.2 | 9 | 0.824 | 31.6 | 9 | 0.028 | 29.4 | 9 |
| 13 | 0.665 | 75.2 | 24 | 2.090 | 47.8 | 8 | 0.612 | 67.4 | 8 | 0.035 | 20.8 | 8 |
| 14 | 0.450 | 10.4 | 26 | 2.297 | 12.3 | 9 | 0.899 | 9.6 | 8 | 0.032 | 13.8 | 9 |
| 15 | 0.451 | 16.8 | 27 | 2.628 | 21.6 | 8 | 0.962 | 16.4 | 8 | 0.038 | 11.5 | 9 |
| 16 | 0.498 | 5.6 | 27 | 2.459 | 5.6 | 9 | 0.914 | 6.7 | 8 | 0.034 | 6.4 | 9 |
| Overall  GM | 0.566 | | | 2.454 | | | 0.893 | | | 0.032 | | |
| Between-lab  GCV | 47.4 | | | 10.0 | | | 14.2 | | | 19.6 | | |
| Overall  GM* | 0.493 | | | 2.447 | | | 0.871 | | | 0.033 | | |
| Between-lab  GCV* | 8.3 | | | 8.4 | | | 6.6 | | | 11.5 | | |

Key:

GM – geometric mean

GCV – geometric coefficient of variation (%)

N – number of estimates used in calculation of mean

*excludes laboratories 2, 3, 4 and 13

Table S4 Relative potencies of samples XC, C1, C2 and C3 relative to candidate 10/198 (S1) in assays using MSP-3 as coating antigen

| **Lab** | **XC** | | | **C1** | | | **C2** | | | **C3** | | |
| --- | --- | --- | --- | --- | --- | --- | --- | --- | --- | --- | --- | --- |
|  | **GM** | **GCV** | **N** | **GM** | **GCV** | **N** | **GM** | **GCV** | **N** | **GM** | **GCV** | **N** |
| 1 | 0.620 | 24.8 | 26 | 3.219 | 17.2 | 9 | 0.807 | 10.9 | 9 | 0.038 | 18.1 | 9 |
| 2 | 2.462 | 174.7 | 21 | 3.224 | 47.6 | 8 | 0.692 | 47.2 | 8 | 0.043 | 25.0 | 8 |
| 3 | 0.575 | 22.7 | 11 | 2.210 | 31.4 | 5 | 1.021 | 11.4 | 5 | 0.065 | 7.6 | 3 |
| 4 | 1.305 | 56.6 | 4 | . | . | . | . | . | . | 0.031 | 31.1 | 2 |
| 5 | 0.547 | 16.8 | 27 | 2.916 | 15.8 | 9 | 0.806 | 13.0 | 9 | 0.047 | 34.2 | 9 |
| 6 | 0.540 | 19.5 | 27 | 3.094 | 13.5 | 9 | 0.889 | 17.3 | 8 | 0.035 | 25.8 | 9 |
| 7 | 0.499 | 25.2 | 27 | 3.249 | 26.6 | 9 | 0.831 | 14.3 | 8 | 0.036 | 26.6 | 9 |
| 8 | 0.530 | 19.0 | 25 | 3.283 | 15.2 | 7 | 0.792 | 8.5 | 9 | 0.039 | 18.4 | 8 |
| 9 | 0.499 | 16.1 | 26 | 3.046 | 16.0 | 8 | 0.769 | 19.2 | 9 | 0.036 | 18.5 | 9 |
| 10 | 0.545 | 47.4 | 21 | 3.172 | 35.4 | 9 | 0.756 | 36.9 | 6 | 0.039 | 43.3 | 7 |
| 11 | 0.569 | 23.5 | 26 | 2.715 | 25.5 | 9 | 0.842 | 22.0 | 9 | 0.053 | 59.7 | 9 |
| 12 | 0.638 | 33.4 | 26 | 2.940 | 18.1 | 8 | 0.745 | 34.1 | 9 | 0.041 | 18.9 | 9 |
| 13 | 1.070 | 75.5 | 24 | 3.516 | 12.2 | 6 | 0.741 | 64.6 | 7 | 0.073 | 96.3 | 6 |
| 14 | 0.543 | 10.0 | 27 | 3.021 | 12.0 | 9 | 0.835 | 17.0 | 9 | 0.041 | 7.6 | 8 |
| 15 | 0.511 | 12.9 | 27 | 2.783 | 13.8 | 9 | 0.852 | 8.8 | 9 | 0.048 | 14.6 | 9 |
| 16 | 0.551 | 6.5 | 25 | 2.937 | 5.0 | 9 | 0.832 | 8.7 | 9 | 0.045 | 9.3 | 9 |
| Overall  GM | 0.665 | | | 3.006 | | | 0.811 | | | 0.043 | | |
| Between-lab  GCV | 55.6 | | | 11.5 | | | 9.5 | | | 25.5 | | |
| Overall  GM* | 0.548 | | | 3.026 | | | 0.812 | | | 0.041 | | |
| Between-lab  GCV* | 7.9 | | | 6.2 | | | 5.4 | | | 13.9 | | |

Key:

GM – geometric mean

GCV – geometric coefficient of variation (%)

N – number of estimates used in calculation of mean

*excludes laboratories 2, 3, 4 and 13

Table S5 EC_50_ estimates for samples XC, C1, C2 and C3 in assays using AMA-1 as coating antigen

| **Lab** | **XC** | | | **C1** | | | **C2** | | | **C3** | | |
| --- | --- | --- | --- | --- | --- | --- | --- | --- | --- | --- | --- | --- |
|  | **GM** | **GCV** | **N** | **GM** | **GCV** | **N** | **GM** | **GCV** | **N** | **GM** | **GCV** | **N** |
| 1 | 0.050 | 48.5 | 24 | 0.012 | 69.0 | 8 | 0.051 | 43.6 | 7 | 2.899 | 37.1 | 9 |
| 2 | 0.077 | 167.7 | 24 | 0.077 | 122.6 | 7 | 0.076 | 132.5 | 9 | 13.398 | 42.7 | 9 |
| 3 | 0.891 | 464.6 | 9 | 0.135 | 179.8 | 3 | 0.320 | 2.8 | 2 | 24.912 | . | 1 |
| 4 | 0.008 | 313.7 | 4 | . | . | . | . | . | . | 1.726 | 79.0 | 4 |
| 5 | 0.032 | 41.7 | 26 | 0.008 | 41.6 | 9 | 0.037 | 18.9 | 8 | 1.416 | 41.3 | 9 |
| 6 | 0.078 | 54.9 | 27 | 0.015 | 30.1 | 9 | 0.059 | 36.1 | 9 | 4.124 | 55.1 | 9 |
| 7 | 0.073 | 14.2 | 27 | 0.017 | 18.1 | 9 | 0.070 | 14.8 | 9 | 3.903 | 10.4 | 9 |
| 8 | 0.054 | 12.0 | 25 | 0.013 | 15.8 | 8 | 0.058 | 18.2 | 9 | 3.052 | 15.0 | 9 |
| 9 | 0.062 | 38.5 | 27 | 0.015 | 47.6 | 9 | 0.057 | 48.3 | 9 | 3.940 | 47.7 | 9 |
| 10 | 0.359 | 43.8 | 21 | 0.058 | 24.6 | 5 | 0.315 | 80.5 | 7 | 19.295 | 28.2 | 5 |
| 11 | 0.063 | 18.3 | 27 | 0.016 | 20.3 | 9 | 0.063 | 17.5 | 9 | 3.409 | 17.7 | 9 |
| 12 | 0.063 | 19.1 | 27 | 0.015 | 28.9 | 9 | 0.069 | 17.8 | 8 | 3.801 | 26.7 | 9 |
| 13 | 0.072 | 91.1 | 26 | 0.047 | 59.8 | 9 | 0.153 | 59.1 | 9 | 4.514 | 22.8 | 8 |
| 14 | 0.048 | 16.7 | 27 | 0.011 | 22.1 | 9 | 0.049 | 11.8 | 9 | 2.522 | 11.6 | 9 |
| 15 | 0.096 | 37.2 | 27 | 0.022 | 49.0 | 9 | 0.084 | 35.0 | 9 | 4.631 | 31.2 | 9 |
| 16 | 0.085 | 7.6 | 27 | 0.021 | 7.9 | 9 | 0.082 | 6.3 | 9 | 4.556 | 11.5 | 9 |
| Overall  GM | 0.073 | | | 0.022 | | | 0.081 | | | 4.492 | | |
| Between-lab  GCV | 170.8 | | | 124.3 | | | 89.0 | | | 120.0 | | |
| Overall  GM* | 0.071 | | | 0.016 | | | 0.069 | | | 3.849 | | |
| Between-lab  GCV* | 79.7 | | | 62.4 | | | 69.5 | | | 82.7 | | |

Key:

GM – geometric mean

GCV – geometric coefficient of variation (%)

N – number of estimates used in calculation of mean

*excludes laboratories 2, 3, 4 and 13

Table S6 EC_50_ estimates for samples XC, C1, C2 and C3 in assays using MSP-1_42_ as coating antigen

| **Lab** | **XC** | | | **C1** | | | **C2** | | | **C3** | | |
| --- | --- | --- | --- | --- | --- | --- | --- | --- | --- | --- | --- | --- |
|  | **GM** | **GCV** | **N** | **GM** | **GCV** | **N** | **GM** | **GCV** | **N** | **GM** | **GCV** | **N** |
| 1 | 0.279 | 18.0 | 25 | 0.062 | 35.6 | 8 | 0.173 | 23.5 | 9 | 4.054 | 18.0 | 8 |
| 2 | 0.181 | 80.8 | 25 | 0.256 | 43.1 | 9 | 0.146 | 102.5 | 7 | 13.864 | 6.3 | 9 |
| 3 | 1.503 | 54.0 | 8 | 0.281 | 106.1 | 3 | 1.062 | 60.7 | 4 | 15.167 | . | 1 |
| 4 | 0.018 | 256.9 | 4 | . | . | . | . | . | . | 1.176 | 78.7 | 3 |
| 5 | 0.323 | 216.2 | 26 | 0.053 | 225.6 | 8 | 0.173 | 195.2 | 9 | 4.280 | 218.5 | 8 |
| 6 | 0.249 | 32.6 | 26 | 0.046 | 33.0 | 9 | 0.112 | 24.4 | 8 | 4.098 | 47.2 | 9 |
| 7 | 0.283 | 15.2 | 27 | 0.048 | 25.3 | 9 | 0.153 | 30.1 | 9 | 4.007 | 9.1 | 9 |
| 8 | 0.220 | 20.0 | 26 | 0.049 | 11.4 | 9 | 0.125 | 18.0 | 8 | 2.960 | 11.6 | 9 |
| 9 | 0.297 | 39.8 | 27 | 0.057 | 54.6 | 9 | 0.159 | 46.0 | 8 | 4.450 | 29.0 | 9 |
| 10 | 1.053 | 31.7 | 23 | 0.189 | 39.6 | 7 | 0.600 | 42.0 | 7 | 13.113 | 43.8 | 8 |
| 11 | 0.294 | 32.4 | 24 | 0.062 | 29.5 | 7 | 0.151 | 17.1 | 8 | 3.917 | 34.0 | 9 |
| 12 | 0.239 | 36.4 | 26 | 0.060 | 12.8 | 9 | 0.152 | 33.5 | 9 | 4.817 | 15.4 | 9 |
| 13 | 0.299 | 85.5 | 24 | 0.109 | 57.9 | 8 | 0.338 | 69.9 | 8 | 5.278 | 47.3 | 8 |
| 14 | 0.202 | 18.8 | 26 | 0.040 | 13.5 | 9 | 0.102 | 8.8 | 8 | 2.774 | 33.2 | 9 |
| 15 | 0.371 | 35.6 | 27 | 0.066 | 48.2 | 8 | 0.184 | 45.7 | 8 | 4.436 | 33.5 | 9 |
| 16 | 0.331 | 8.2 | 27 | 0.067 | 7.5 | 9 | 0.184 | 12.8 | 8 | 4.686 | 6.7 | 9 |
| Overall  GM | 0.276 | | | 0.077 | | | 0.197 | | | 4.762 | | |
| Between-lab  GCV | 150.4 | | | 88.4 | | | 89.4 | | | 89.5 | | |
| Overall  GM* | 0.309 | | | 0.061 | | | 0.167 | | | 4.406 | | |
| Between-lab  GCV* | 52.8 | | | 48.1 | | | 56.0 | | | 46.6 | | |

Key:

GM – geometric mean

GCV – geometric coefficient of variation (%)

N – number of estimates used in calculation of mean

*excludes laboratories 2, 3, 4 and 13

Table S7 EC_50_ estimates for samples XC, C1, C2 and C3 in assays using MSP-3 as coating antigen

| **Lab** | **XC** | | | **C1** | | | **C2** | | | **C3** | | |
| --- | --- | --- | --- | --- | --- | --- | --- | --- | --- | --- | --- | --- |
|  | **GM** | **GCV** | **N** | **GM** | **GCV** | **N** | **GM** | **GCV** | **N** | **GM** | **GCV** | **N** |
| 1 | 0.498 | 23.6 | 26 | 0.098 | 32.7 | 9 | 0.407 | 35.6 | 9 | 7.909 | 30.1 | 9 |
| 2 | 0.304 | 110.5 | 21 | 0.375 | 36.1 | 8 | 0.468 | 126.3 | 8 | 28.558 | 3.1 | 8 |
| 3 | 2.806 | 48.3 | 11 | 0.543 | 75.7 | 5 | 1.732 | 33.2 | 5 | 27.632 | 17.4 | 3 |
| 4 | 0.046 | 111.5 | 4 | . | . | . | . | . | . | 1.761 | 27.8 | 2 |
| 5 | 0.269 | 59.1 | 27 | 0.046 | 22.5 | 9 | 0.234 | 60.6 | 9 | 2.663 | 84.7 | 9 |
| 6 | 0.565 | 23.5 | 27 | 0.105 | 24.7 | 9 | 0.345 | 10.7 | 8 | 8.304 | 40.2 | 9 |
| 7 | 0.632 | 24.6 | 27 | 0.085 | 44.5 | 9 | 0.463 | 46.0 | 8 | 7.710 | 24.3 | 9 |
| 8 | 0.571 | 18.3 | 25 | 0.078 | 115.5 | 7 | 0.383 | 29.9 | 9 | 6.926 | 16.3 | 8 |
| 9 | 0.608 | 34.8 | 26 | 0.087 | 28.6 | 8 | 0.438 | 40.9 | 9 | 8.994 | 46.2 | 9 |
| 10 | 1.831 | 34.5 | 21 | 0.303 | 123.1 | 9 | 1.263 | 19.4 | 6 | 22.216 | 31.1 | 7 |
| 11 | 0.685 | 19.7 | 26 | 0.142 | 26.9 | 9 | 0.453 | 21.6 | 9 | 8.452 | 15.9 | 9 |
| 12 | 0.513 | 35.5 | 26 | 0.118 | 9.4 | 8 | 0.427 | 17.1 | 9 | 7.906 | 20.9 | 9 |
| 13 | 0.650 | 105.7 | 24 | 0.158 | 122.3 | 6 | 0.902 | 108.4 | 7 | 11.176 | 18.2 | 6 |
| 14 | 0.394 | 8.9 | 27 | 0.069 | 12.0 | 9 | 0.264 | 5.7 | 9 | 5.116 | 13.1 | 8 |
| 15 | 1.025 | 28.5 | 27 | 0.194 | 23.5 | 9 | 0.647 | 25.7 | 9 | 10.040 | 26.9 | 9 |
| 16 | 0.745 | 5.2 | 25 | 0.138 | 6.9 | 9 | 0.509 | 7.2 | 9 | 9.120 | 8.6 | 9 |
| Overall  GM | 0.555 | | | 0.134 | | | 0.509 | | | 8.607 | | |
| Between-lab  GCV | 142.9 | | | 95.4 | | | 71.9 | | | 107.9 | | |
| Overall  GM* | 0.620 | | | 0.108 | | | 0.441 | | | 7.892 | | |
| Between-lab  GCV* | 60.6 | | | 63.9 | | | 53.6 | | | 62.0 | | |

Key:

GM – geometric mean

GCV – geometric coefficient of variation (%)

N – number of estimates used in calculation of mean

*excludes laboratories 2, 3, 4 and 13
